# Leiomyoma of the mandible in a child

**DOI:** 10.4103/0973-029X.80015

**Published:** 2011

**Authors:** BVR Reddy, B Shoba Rani, CH Anuradha, P Chandrasekhar, R Shamala, KP Lingamaneni

**Affiliations:** *Department of Oral Pathology, SIBAR Institute of Dental Sciences, Takkellapadu, Guntur, Andhra Pradesh, India*

**Keywords:** Immunohistochemistry, leiomyoma, smooth muscle tumor

## Abstract

Leiomyomas are the benign tumors of the smooth muscle that usually arise in the gastrointestinal system and in the uterus. Oral leiomyomas are uncommon due to the paucity of the smooth muscles in the mouth (except in blood vessels) and those of the mandible are extremely rare. Leiomyomas have been classified as solid, angiomyoma (vascular leiomyoma), and epithelioid variants. Here, we report a rare case of leiomyoma of the mandible in a 9-year-old child, together with conventional histopathologic and immunohistochemical findings.

## INTRODUCTION

Leiomyoma was first reported by Blanc in 1884.[1] Leiomyoma is a benign tumor of smooth muscle origin, which is usually diagnosed in the gastrointestinal tract, uterus and skin.[1–4] It most commonly arises in the retroperitoneum, mesentry, omentum or subcutaneous and deep tissues of the limbs.[5] It is found uncommonly in the oral cavity because of the paucity of the smooth muscles in this region (except in the blood vessel walls).[1] If at all it occurs, it shows its predilection in the posterior portion of the tongue, lips, palate, cheeks, gingiva and salivary glands.[6] Intraosseous oral leiomyomas are rare and those of mandible are very rare.[3] The origin of oral leiomyomas is smooth muscle of vessel walls, the circumvallate papilla and atypical arrectores pilorum muscles in the cheek.[7] Clinically, they can be seen at any age ranging from infancy to 76 years, but mostly seen in middle-aged group[6,7] and exhibits male predilection.[8] Radiographically, angioleiomyomas manifest as unilocular or multilocular radiolucent lesions with either an ill-defined or a well-defined sclerotic border. Cortical expansion of the alveolar plates may be evident. Root resorption has also been shown.[4] Histopathologically, it is composed of spindle cells arranged in whorled and interlaced fascicular pattern. The cells show elongated nuclei with fusiform or blunt ends.[2] The line of treatment is conservative surgical excision.[1,2]

## CASE REPORT

**Figure 1:**
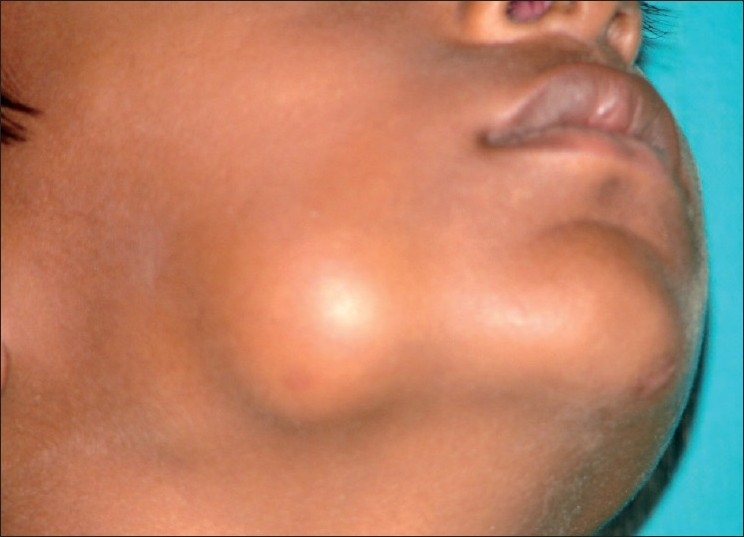
Extraoral swelling on the right body of the mandible

A 9-year-old boy reported to the Department of Oral and Maxillofacial Pathology, SIBAR Institute of Dental sciences, with an asymptomatic extraoral swelling on the right body of the mandible, which had been evident for 2 months [Figure 1]. According to the patient, the swelling was initially small and gradually progressed to the present size and was asymptomatic. His past medical history and family history were noncontributory. On clinical examination, right facial asymmetry was observed. A solitary, extraoral swelling was seen on the right side of the body of the mandible, which was oval in shape and was 4 × 3.5 cm in size, extending anteriorly 2 cm away from the corner of the mouth and posteriorly 1 cm in front of the angle of the mandible. Superiorly, the lesion was extending up to the line joining the corner of the mouth and the tragus of the ear and inferiorly to the lower border of the mandible. It was hard and nontender on palpation [Figure 1]. Intraorally, the overlying mucosa was intact and smooth and there was no tooth mobility. The regional lymph nodes were not enlarged. The clinical differential diagnosis including central ossifying fibroma, central giant cell granuloma, neurofibroma, neurilemmoma, myofibroma of the mandible and osteoma was considered.

**Figure 2:**
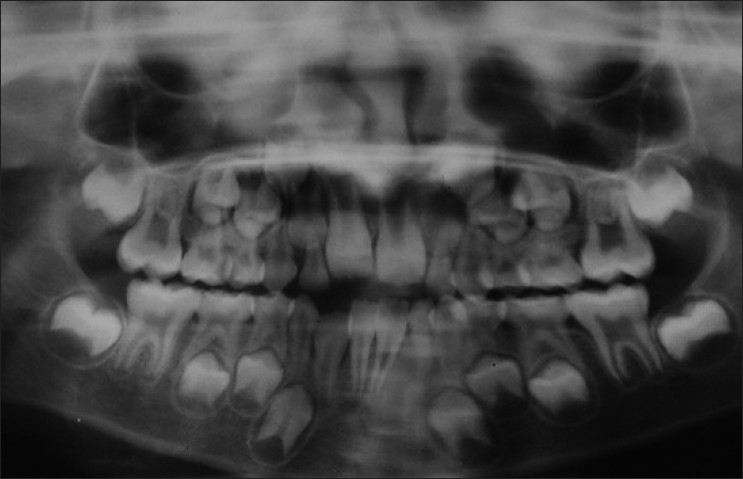
Panoramic radiograph revealing faint periosteal reaction in the vicinity of permanent first molar and developing tooth buds of right canine and premolars

**Figure 3:**
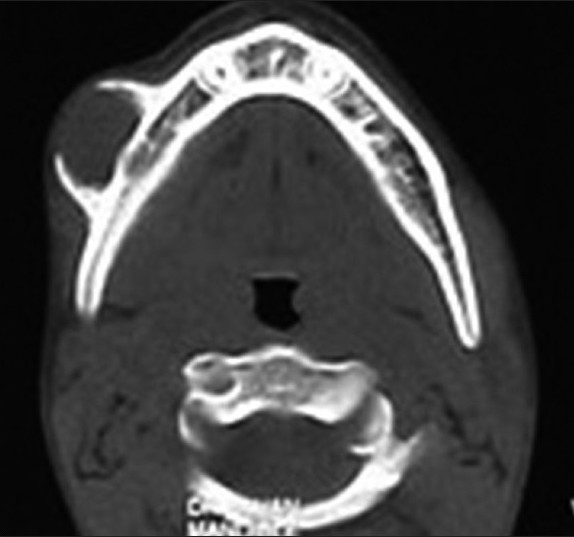
The axial CT view in bone window showing a massive periosteal reaction on the buccal surface, demonstrating the sclerotic borders of the lesion with soft tissue density within the central zone

On radiographic investigations, the panoramic radiograph of the right mandibular premolar-molar region showed faint periosteal reaction in the vicinity of permanent first molar. The developing tooth buds of right canine and premolars appeared to be uninfluenced by the pathosis in the region as compared to the left side. The periosteal bone reaction was noted [Figure 2]. As the panoramic radiograph revealed no significant findings, an axial computed tomography (CT) was advised. The axial CT view in bone window at the level of inferior border of the mandible showed a massive periosteal reaction on the buccal surface, demonstrating the sclerotic borders of the lesion. The lesion appears to have soft tissue density within the central zone as seen from this window, suggesting that the lesion is of soft tissue region [Figure 3]. A radiographic differential diagnosis of neurofibroma, neurilemmoma, myofibroma, desmoplastic fibroma of the mandible, adenomatoid odontogenic tumor, periosteal osteoblastoma and periosteal osteosarcoma were suggested. A provisional diagnosis of myofibroma of the mandible was given, based on the clinical and radiographic findings.

**Figure 4:**
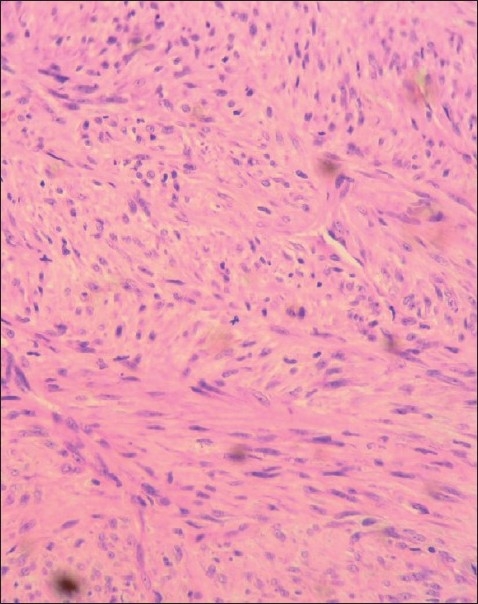
Spindle cells arranged in interlaced fascicular pattern, showing elongated nuclei with blunt ends

The lesion was removed under general anesthesia via an extraoral approach. A well-circumscribed lesion was observed and the lesion was easily removed from the surrounding tissues. Macroscopically, the lesion was roughly a spherical mass, brownish white in color, measuring 1 × 1 × 1 cm and was firm in consistency. Microscopically, the tissue section exhibited cellular areas intermingled with collagenous and hyalinized areas. The cells were predominantly spindle shaped and showed elongated nuclei with fusiform or blunt ends [Figure 4]. The tissue also exhibited numerous vascular areas. In some areas, the cells along with the collagen fibers were arranged in fascicles. The periphery of the lesion exhibited spicules of vital reactive bone. Most of the trabeculae were perpendicularly arranged with respect to the periphery of the lesion. There was a minimal diffuse chronic inflammatory cell infiltrate. Histopathologically, a diagnosis of spindle cell neoplasm was given.

**Figure 5:**
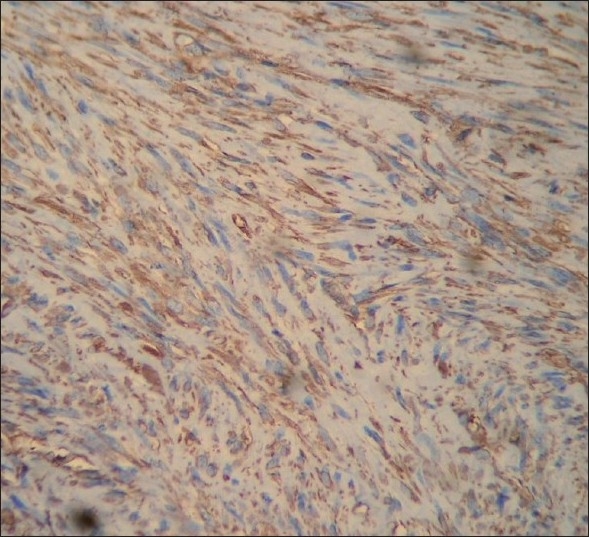
The tumor was positive for vimentin

**Figure 6:**
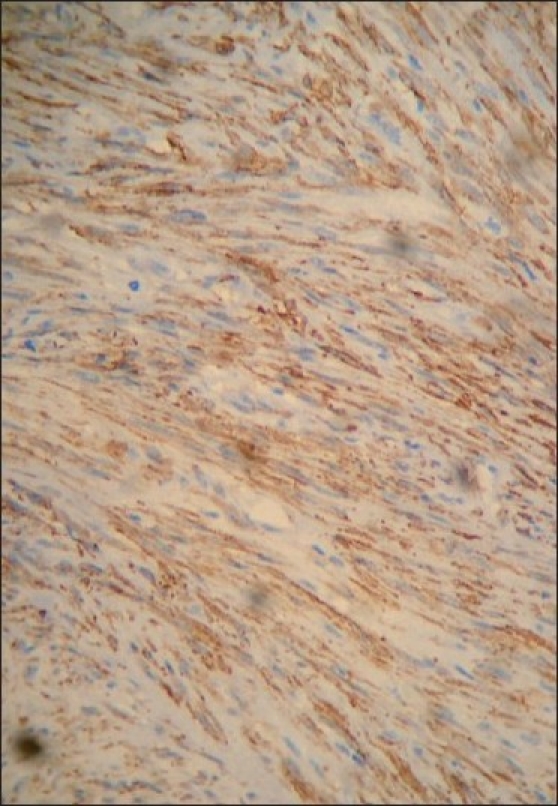
The tumor was positive for α-smooth muscle actin

As all the spindle cell neoplasms show similar histopathologic features, immunohistochemistry (IHC) was suggested. IHC was performed against vimentin, smooth muscle actin and S-100 protein. The tumor was positive for α-smooth muscle actin [Figure 5] and vimentin [Figure 6] and negative for S-100 protein and desmin. Based on the IHC report, a confirmatory diagnosis of leiomyoma was given. No complications were observed during the consecutive postoperative sessions in past 3 years and perfect bone healing occurred without any recurring pathoses.

## DISCUSSION

The occurrence of leiomyoma is rare in the oral cavity. It accounts for 0.42% of all soft tissue lesions in the oral cavity.[9] Intraosseous leiomyomas are very rare.[1,6,9,10] A review of the English language literature showed that 10 cases have been described.[1]

Huseyin Koca *et al*, reported that they can be seen at any age ranging from infancy to 75 years and are most commonly seen in the middle-aged group.[6,7] It exhibits slight predilection for males.[8] The most common sites of oral leiomyoma are posterior portion of the tongue, lips, palate, cheeks, gingiva and salivary glands.[6] Intraosseous lesions are rare, and if at all they occur, show predilection for mandibular posterior region with cortical involvement.[6] The rarity in the mandible could be explained by the small amount of smooth muscle in the mandible. The vascular walls may be the only source of smooth muscle in the mandible, but heterotopic embryonal tissue also has been suggested.[1,3] In our case, the age, gender and site predilection were consistent with the earlier reports.

Radiographically, angioleiomyomas manifest as unilocular or multilocular radiolucent lesions with either an ill-defined or a well-defined sclerotic border. Cortical expansion of the alveolar plates may be evident. Root resorption has also been shown.[4] In our case, the panoramic radiograph revealed no characteristic findings, and hence, an axial CT was advised.

According to World Health Organization, leiomyomas are classified into three histological groups: (a) vascular (angioleiomyoma), accounts for 74% of the cases; (b) solid, accounts for 25% of the cases; (c) epithelioid (leiomyoblastomas), accounts for less than 1% of the cases.[5,10] Histologically, the tumor is composed of spindle cells arranged in a whorled and interlaced fascicular pattern. The cells show elongated nuclei with fusiform or blunt ends, and perinuclear vacuolization can sometimes be noted. Mature smooth muscle cells have the distinctive characteristics of small and uniform nucleus and broad eosinophilic cytoplasm.[1,2] In our case, the histopathologic findings were in accordance with the above-mentioned features of angioleiomyoma.

The histopathologic diagnosis of leiomyoma is relatively difficult because of the resemblance to many other tumors with spindle-shaped cells. The differential diagnosis would include myofibroma, hemangiopericytoma, neurofibroma, neurilemmoma, nodular fasciitis, fibrous histiocytoma and schwannoma.[3,4]

Immunohistochemically, leiomyomas are reactive with vimentin, desmin, α-smooth muscle actin and muscle-specific actin. In our case, immunohistochemical findings revealed positivity for vimentin, smooth muscle actin and negativity for S-100 protein. These features helped us to exclude fibroblastic and neural tumors while indicating myogenic differentiation of the tumor.

The malignant transformation rate has not been reported in the literature.[2] In general, from clinicoradiographic data, the tumor doubling time and the resorption of surrounding areas are suggestive of malignancy. From the histologic point of view, the mitotic count has been considered as the most important parameter for differentiating benign and malignant smooth muscle tumors. In our case, though there was rapid increase in size, the tumor had minimal mitotic count which suggests that our case is benign. Despite these findings, it must be remembered that some benign lesions have been metastasized during the later life.[1] The recurrence rate is very low.[2] The line of treatment is conservative surgical excision.[1,2,6]

## CONCLUSION

In summary, leiomyoma in the oral cavity is rare and is still a rarer entity in mandible due to paucity of the smooth muscles. But it should be considered in the clinical differential diagnosis in the pediatric patients due to its wide age range of occurrence. Even though in the present case, clinicoradiographic and histologic findings favored a benign process, a careful periodic evaluation should be planned as benign lesions have metastasized after latency period.
